# Cryopreservation of ovarian tissue – what's known so far and future perspectives

**DOI:** 10.3389/frph.2026.1768400

**Published:** 2026-03-31

**Authors:** Sabine Eberhart, Hazem Khalifa, Laura Rafensteiner, Josef Lehner, Katharina Hancke, Karin Bundschu

**Affiliations:** Department of Gynaecology and Obstetrics, FePro-Ulm, University Hospital of Ulm, Ulm, Germany

**Keywords:** cryopreservation, cryoprotective agents, nanowarming, ovarian tissue cryopreservation (OTC), slow freezing, vitrification, whole organ freezing

## Abstract

**Background:**

Cryopreservation is widely used across the life sciences to enable long-term storage of living cells and tissues for research or later clinical use. Its core principle is the arrest of biological activity at extremely low temperatures. Ovarian tissue cryopreservation (OTC) has become an important fertility-preserving option for women and prepubertal girls facing gonadotoxic cancer therapies.

**Objective and rationale:**

This review summarizes the history of OTC and provides an overview of current procedures and their relevance for fertility preservation. It outlines key principles of cryopreservation, including different techniques, cryoprotective agents, molecular mechanisms, recent achievements, remaining challenges, and future perspectives. Although OTC is clinically established, protocols remain variable and require further optimization to improve tissue and follicle viability. Differences in media composition, cryoprotectants, slow freezing vs. vitrification, and thawing or warming procedures—along with ongoing debate over which technique is superior—highlight the need for research toward a standardized approach.

**Search methods:**

PubMed and MEDLINE were searched for literature published before June 2025 using the keywords *cryopreservation, ovarian tissue cryopreservation (OTC), vitrification, slow freezing, nanowarming, whole organ freezing, cryoprotective agents*. Reference lists were screened back to 1993. Only English-language publications were included.

**Outcomes:**

The literature review shows that no universally standardized OTC protocols exist for slow freezing or vitrification. Although both methods are routinely applied worldwide, differences in crucial steps may affect tissue quality and clinical outcomes. Thawing and warming, also essential for tissue viability, is not standardized. These findings emphasize the need for continued optimization. Research on whole-organ freezing and nanowarming is also progressing. Nanowarming aims to enable uniform warming of larger, more complex tissues, with two promising technologies—electromagnetic warming and photothermal heating—currently evaluated in animal models.

**Wider implications:**

As oncological treatments advance and more young female cancer patients survive, the demand for effective and standardized OTC procedures continues to grow. OTC remains the preferred fertility-preservation method for patients unable to undergo ovarian stimulation or for prepubertal girls. This review outlines current methods, highlights advances in nanowarming and whole-organ cryopreservation, and provides future perspectives for improving OTC and related technologies.

## Graphical Abstract

**Figure:**
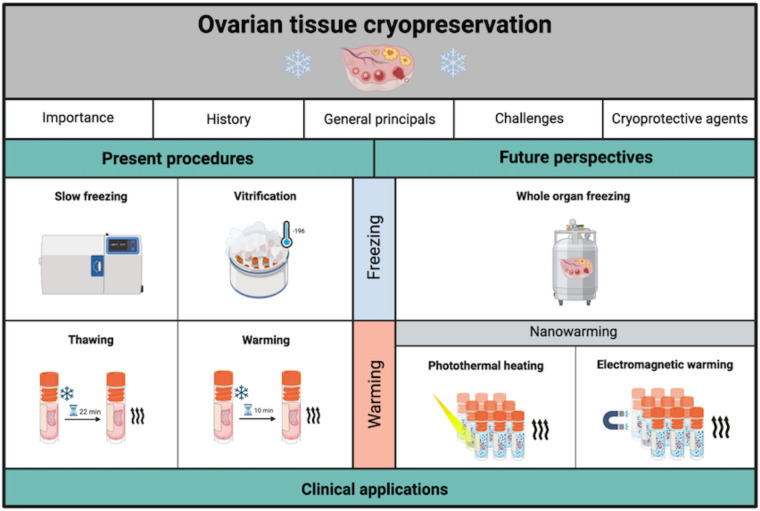
The history, importance, general principals, challenges, and cryoprotectants for ovarian tissue cryopreservation are presented, and established procedures, future perspectives, and their clinical applications are outlined.

## Introduction

Long-term storage of live cells and tissues for further research or later clinical usage is an essential method in many fields of life sciences (1). This routinely used method for retaining biological and biochemical activities of cells is called cryopreservation, and describes the cryogenic storage at temperatures below −150 °C (2). Nevertheless, the process of freezing viable cells/tissues is not free of challenges and obstacles that hinder fulfilling these aims. In order to achieve the purpose of cryopreservation, the frozen cells/tissues need to retain their integrity, viability and functionality after thawing. In other words, they have to function physiologically like fresh cells/tissues. Although cryopreservation techniques were developed and improved significantly during the last century and are currently widely used in many biological and medical applications, the efficacy and outcome have not yet reached the desired level that enables optimal functionality for the rewarmed samples. Basically, cryopreserving single cells is less challenging and more effective than freezing simple homo-cellular tissues (3). Even more challenging is the cryopreservation of composite tissues, or even entire organs.

**Figure 1:**
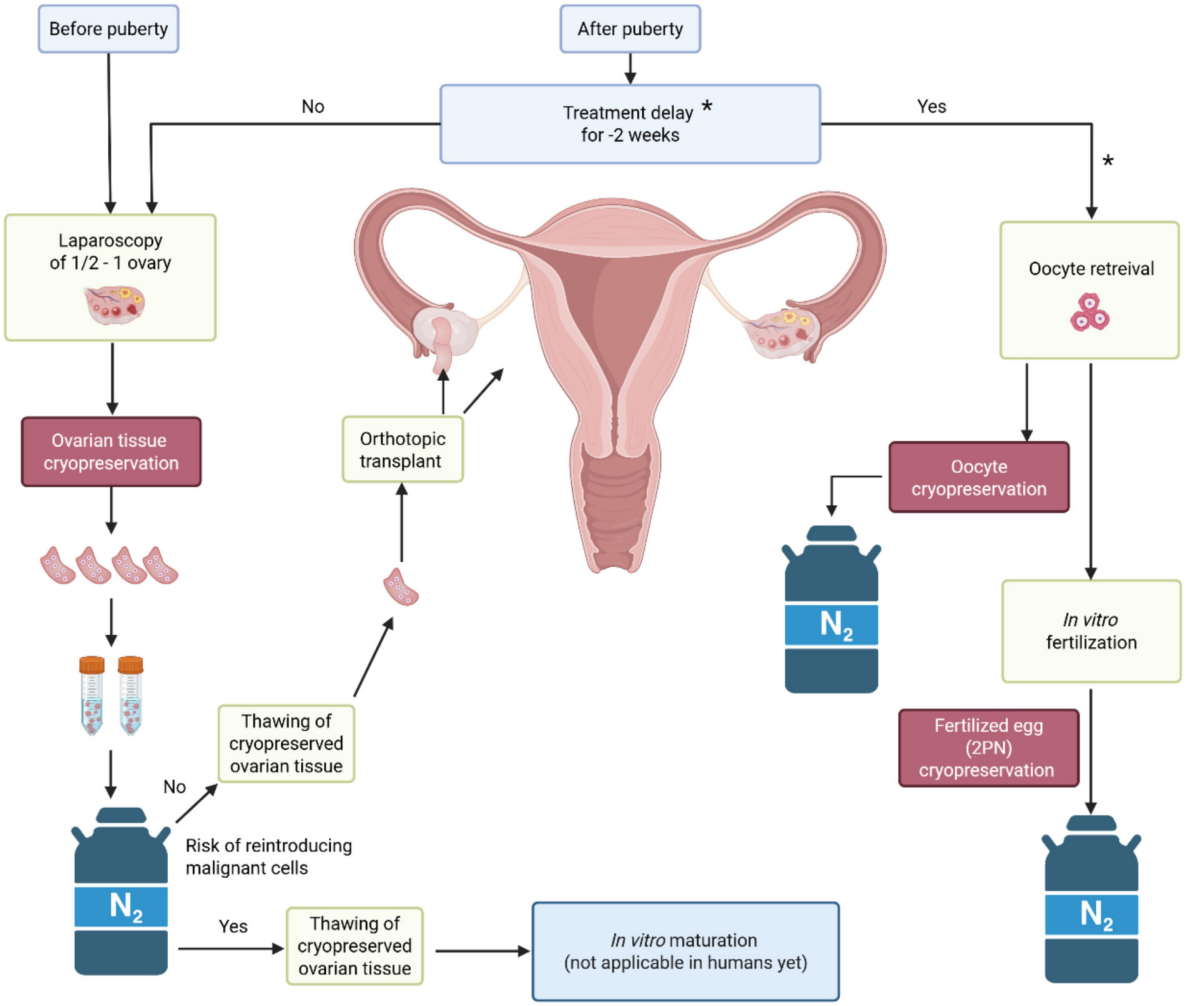
Overview of fertility preservation options. The overview states present options of fertility preservation for women and girls depending on puberty status urgency of gonadotoxic treatment and metastatic risk. *Cryopreservation of oocytes/embryos as fertility preservation option is not part of this review. This option is included in this figure to complete the general decision process and to demonstrate the importance of cryopreservation, especially OTC. Created in BioRender. Eberhart, S. (2026) https://BioRender.com/xr5yjcg.

One of the most important and promising medical application of cryopreservation is the ovarian tissue cryopreservation (OTC) (Figure 1), which nowadays is widely used for fertility preservation in women and prepubertal girls, who are diagnosed with cancer and have to accordingly undergo gonadotoxic chemo- or radiotherapy (4). This review represents the current state of the art in OTC and its application and significance for fertility preservation. We explain the main principles and indicate the most recent achievements and challenges.

## Historical background and development of OTC

**Figure 2:**
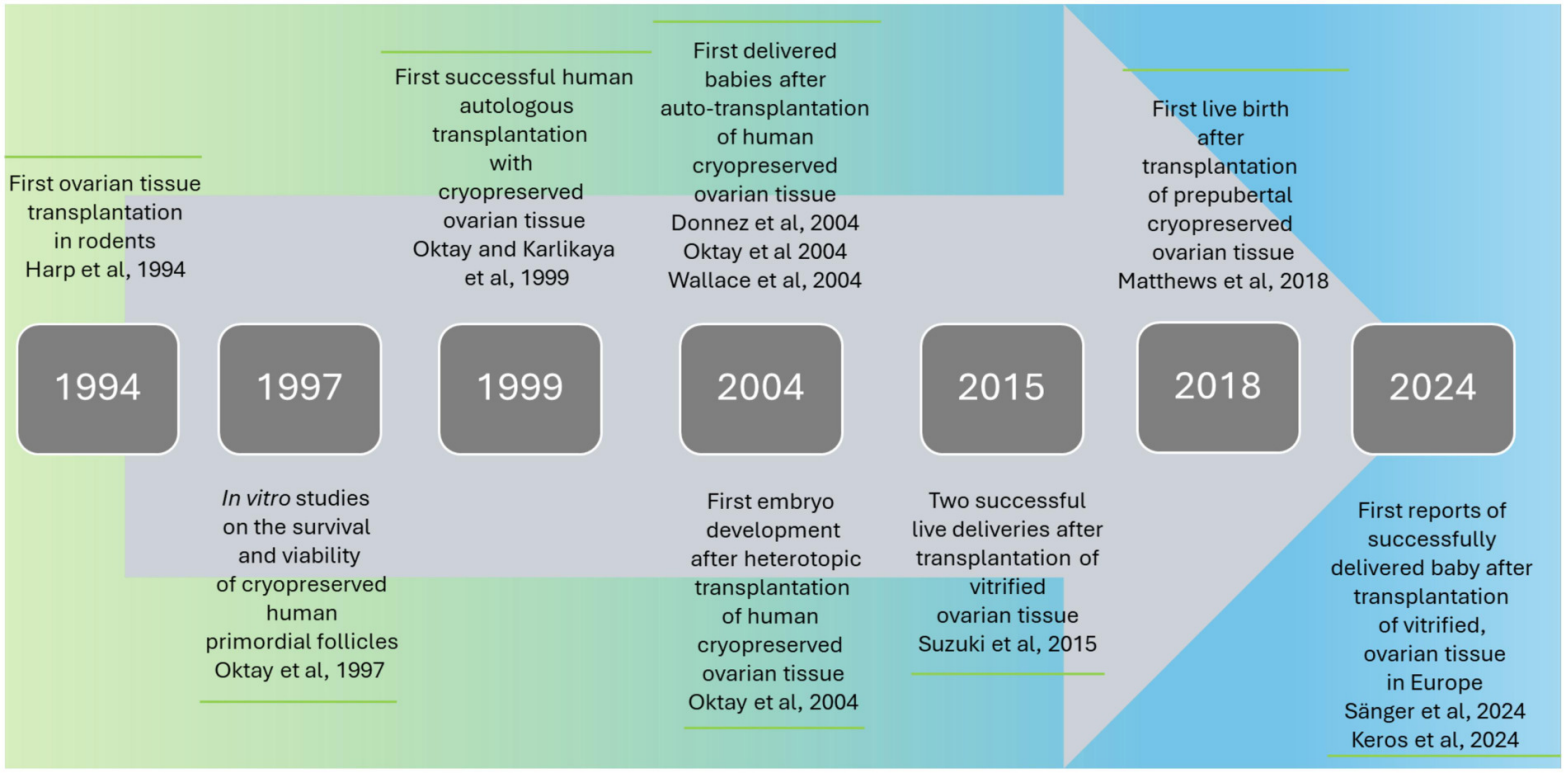
History of key advancements in ovarian tissue cryopreservation (OTC) from 1994 to 2024.

OTC has been offered to patients since more than 20 years (5) to treat infertility caused either by gonadotoxic therapy or by non-cancerous diseases like sickle cell anemia (6, 7). The origin of OTC as allografting (fresh) ovarian tissue goes back to 1906, where a case of restoration of endocrinological function was reported by a New Yorker surgeon (8). Even though there is a report, that this woman delivered a child 4 years after allogenic ovarian tissue transplantation, it is highly unlikely, that this actually happened with an allogenic transplant without any immunosuppressive medication. Due to the lack of suitable cryoprotectants and freezing protocols that could effectively reduce tissue damage during the freezing process, efforts undertaken by researchers on rats in the 1950s were abandoned (9, 10). It took another 20 years to discover alternatives to glycerol as cryoprotectant, and another 20 years to open the door for advancements in the techniques of OTC (11). Harp et al. (12), Cox et al. (13) and Sztein et al. (14) were the first in 1994, 1996 and 1998, respectively, to repeat the OTC experiments in rodents and prove that it was indeed possible to restore the cyclic activity as well as the endocrine function of the ovary with cryopreserved and re-transplanted ovarian tissue (Figure 2). The animal studies, even though successful, also pointed to the large follicle loss by using cortical pieces and waiting for them to be revascularized after transplantation. The ischemic revascularization period causes 68% of primordial follicle loss, whereas during the freezing process only 7% of primordial follicles are lost (15). The first advancements in OTC were reported shortly after the first successful experiments in rodents by using modern cryoprotectants. In 1996, Hovatta et al. published that with DMSO and propanediol-sucrose human primordial follicles survived a cryopreservation of 24 h to 5 weeks (16). These results were supported by Oktay et al. in 1997, stating that the viability rates of human primordial follicles isolated from fresh and cryopreserved tissue were similar (17). Since 1996, several groups have conducted intensive research on cryopreservation of ovarian tissue, including groups of O. Hovatta (16, 18), J. Donnez (6, 19–24), R. Gosden (11, 17), S.J. Silber (25–28), N. Suzuki (29, 30) and K. Oktay (17, 31–36). The latter group published the first successful autologous transplantation of cryopreserved human ovarian tissue (32) and the first embryo development after heterotopic transplantation (34). J. Donnez et al., Oktay et al., and Wallace et al. were the first to publish a live birth after an auto-transplantation of cryopreserved human ovarian tissue (19, 36, 37). Additionally, Suzuki et al. successfully mastered the transplantation of vitrified ovarian tissue, followed by two live deliveries (30). The latest advancements were made in prepubertal patients by Matthews et al. (38) and in the methods used for vitrification in combination with rapid warming by Saenger et al. in 2024 (39). Another successful live birth after transplantation of vitrified ovarian tissue was published in 2024 by Keros et al. (40) (Figure 2).

## Importance of ovarian tissue cryopreservation

The field of oncofertility was established in 2006 by the oncofertility consortium and Teresa K. Woodruff (41). In parallel, FertiPROTEKT was founded in Germany (42) as a cooperation of university- centers and clinics. This network now includes more than 150 centers in Germany, Switzerland and Austria and aims to optimize fertility counseling and treatment (43). This subfield, which bridges oncology and reproductive research demonstrates how important fertility preservation is for cancer patients. Cancer patients are the largest patient group to whom OTC is offered as fertility preservation option. However, OTC is also offered to patients with hemoglobin disorders such as sickle cell disease or beta-thalassemia, as well as to patients with autoimmune diseases like systemic lupus erythematosus, aplastic anemia and multiple sclerosis (44, 45). Patients with Turner syndrome and galactosemia as well as myelodysplastic syndrome also have the possibility to preserve their fertility by OTC (46–48).

Although recovery chances from cancer in adolescents and young adults (AYAs) have been relevantly increased due to improved oncological therapies over the last years, cytotoxic treatments, in particular chemo- and radiotherapies, have gonadotoxic effects on the ovaries, resulting in partial or complete loss of ovarian function and leading to infertility (49, 50). Accordingly, valid fertility preservation methods should be offered and provided to young female cancer patients (51). Currently, the most established option of fertility preservation is the assisted reproductive technology (ART) with controlled ovarian stimulation and trans-vaginal oocyte retrieval prior to cancer therapy. The retrieved metaphase II (MII) oocytes are cryopreserved and stored for later use in intracytoplasmic sperm injection (ICSI), when pregnancy is desired for family planning after recovery (52).

However, ovarian stimulation protocols require quite a long time of two to three weeks, which sometimes contradicts the necessity for the immediate start of chemo- or radiotherapy. Furthermore, pregnancy rates depend on the amount of gained oocytes and age of the patient. Moreover, this option is not suitable for prepubertal girls, as oocytes are not yet sufficiently mature for stimulation (23, 51). Another option is the surgical transposition of the ovaries (oophoropexy) out of the pelvic irradiation field (53–55), but this procedure is not suitable for systemic chemotherapy.

Therefore, in cases of planned gonadotoxic chemotherapy and time-critical start, OTC emerges as a valid alternative option for fertility protection (56). In OTC, usually half or one whole ovary is removed laparoscopically prior to cytotoxic therapy. The ovarian tissue is cryopreserved in small cortical strips. After cancer recovery, these strips are thawed and transplanted either orthotopically on the ovary or as small fragments into a peritoneal pouch (57). OTC is currently the only option for prepubertal girls diagnosed with cancer, as primordial follicle-containing ovarian cortex can be obtained from women at any age (51). Primordial follicles contain immature oocytes, which were found to survive cryopreservation even better than mature oocytes (58). So far, there is only one known case of live birth after taking ovarian tissue for cryopreservation of a prepubertal girl with β-thalassemia and not a single one after gonadotoxic treatment for cancer (38). Up to now, reimplantation of cryopreserved ovarian tissues from postpubertal patients resulted in more than 200 healthy live births worldwide (59). The FertiPROTEKT consortium conducted the worldwide largest case study, in which 21 pregnancies were recorded and 17 babies were delivered after the transplantation of 95 ovarian tissues (60). Comparing to cryopreservation of oocytes and following ART, OTC can restore not only ovarian gametogenic functions, but also steroidogenic hormonal functions (4), which substitutes for pharmacological hormonal replacement therapies (HRTs) after loss of ovarian endocrine functions due to cancer treatment. This was supported in 2013, when 93% of patients were reported to restore ovarian endocrine function after 4.7 months of reimplanting cryopreserved ovarian tissues (24). A key limitation of using ovarian tissue strips to restore ovarian endocrine function is that the effect is temporary and the duration of endocrine activity varies considerably from patient to patient (61). Another key limitation of retransplanting ovarian tissue is the risk of reintroducing malignant cells (62). However, OTC can also be used for fertility preservation in patients with non-malignant diseases, like endometriosis, autoimmune diseases (38, 39), or prior to stem-cell transplantation in sickle cell anemia or thalassemia major (63).

Although OTC is now an established treatment (64), cryopreservation protocols still offer potential for optimization in terms of tissue and follicle viability—evaluated for example by neutral red or Calcein-AM staining. The published protocols differ regarding the composition of media and cryoprotective agents, the use of slow freezing vs. vitrification methods and the thawing or warming protocols. The differences between the protocols and the ongoing discussions whether slow freezing or vitrification is the better cryopreservation method, reveals that there is still need for further research in order to optimize these protocols and find a “gold standard”.

## General principles and challenges of cryopreservation

Water is the most abundant matter in live cells, composing more than 70% of the total cell mass, beside organic molecules and inorganic ions (65). Exposing live cells to very low temperatures lead the cellular water content to freeze spontaneously, forming intra- and extracellular small ice nuclei. These nuclei act as centers to nucleate fast-growing ice crystals by binding the surrounding liquid water molecules (66). This results in two major lethal effects on the cell. First, the spontaneously growing ice crystals increase in size and decrease in density, causing mechanical stress on the outer and intracellular membranes, which leads to disruption of these membranes and thus to cell death. Second, intracellular organic and inorganic solutes are excluded from the forming ice crystals. As the liquid water fraction decreases relatively to the solid fraction, the solutes concentrations elevate within the residual liquid fraction to a lethal hypertonic concentration, causing cells to die by osmotic stress (2, 67). In addition to the effects of the ice crystal, slow freezing and thawing cells induce lipid peroxidation by molecular oxygen (68, 69), which results in oxidative injuries, e.g., membrane damage, DNA fragmentation (70) and activation of programmed cell death (71, 72).

For single cells and cell suspensions, cryopreservation allows equal exposure to the low temperatures for all cells and at all surfaces of each cell and hence provides a uniform cooling rate for the whole sample. However, heterogenous tissues are composed of various types of cells, which have different tolerances for freezing processes (3). Moreover, these cells are arranged in multilayered 3-dimensional (3D) architecture, held by intercellular matrices and accordingly, are not equally exposed to low temperatures. As a result, some cells cool down and freeze faster than others, which is followed by stress and higher degrees of damage. Nevertheless, cryopreservation is routinely applied for different types of tissues, e.g., skin, cartilage, blood vessels, corneas and nervous tissues (3), in addition to OTC, which is also routinely applied and continuously improved.

## Cryoprotective agents

In order to reduce these lethal effects of cryopreservation, cryoprotective agents (CPAs), also known as cryoprotectants, are added to the cryopreservation medium. CPAs are inorganic and organic solutes, which allow higher post-thawing recovery (2). Generally, there are two main categories of CPAs. The first category includes the non-permeating agents (NPAs), which are large covalently linked molecular polymers (73). The second category includes the permeating agents (PAs), which are much smaller non-ionic molecules that can penetrate through the cellular membrane (74). Although CPAs mitigate the harmful effects caused by ice crystal formation, they exhibit toxic effects to the cells, especially PAs, as they penetrate into the cell and interact with the cellular components (2). Theoretically, cells, tissues and even organs could be cryopreserved without any ice crystal formation, if unlimited high concentrations of CPAs could be applied (75). However, this is practically not applicable, because high concentrations of CPAs are toxic to viable cells, and the time between adding the CPA and the complete cease of metabolism by freezing is enough to damage all cells. This is also the case for the time between thawing the cells and removing the CPA. Therefore, CPAs should be added in limited concentrations just before freezing and should be removed immediately after thawing, to minimize the time the cells are exposed to their toxic effects. Only viable cells can suffer from injuries and stress. Once a cell reaches the cryogenic status, it is not considered as a live system anymore and is therefore not affected by toxicity. Hence, the main challenging harms are the exposure times between adding CPAs and reaching the cryogenic status during freezing, and between leaving cryogenic status and eliminating CPAs after thawing. The shorter the exposure time during these two phases, the better the recovery after thawing (76).

### Non-permeating cryoprotective agents (NPAs)

In contrast to permeating agents, non-permeating cryoprotective agents consist of larger solutes, which usually occur in dimers, trimers or polymers. Consequently, they are unable to penetrate through biological membranes and accordingly, they accumulate outside the cells and elevate the extracellular osmotic pressure (77), resulting in dehydrating the cells and reducing the intracellular water content that forms ice crystals during the cooling process. Usually, a gradual addition of NPAs should be coupled with a programmed freezing system to achieve a balance between dehydration rate and cooling rate, to avoid ice crystal formation, as well as to minimize the exposure to dehydration injuries (78). Commonly used non permeating agents are polyethylene glycol (PEG), raffinose, sucrose, polyvinylpyrrolidone (PVP) and trehalose (79).

### Permeating cryoprotective agents (PAs)

Glycerol, ethylene glycol, dimethyl sulfoxide (DMSO), and 1,2-propandiol are the most commonly used permeating agents. They have low molecular weights and are highly soluble in water, which allow them to pass through the cell membrane (80). DMSO, which is the most widely used cryoprotective agent, causes the formation of water pores in biological membranes at concentrations around 10%. This facilitates the replacement of water by the cryoprotectants and therefore accelerates the vitrification (67, 81). The protective effect of the permeating agents is based on their ability to bind intracellular free water molecules and accordingly, block their availability to form ice crystals, as only free non-bound water molecules can participate in ice crystals via forming hydrogen bonds with ice nucleus molecules (74).

In 2019, Ellen Rivas Leonel et al. (80) reviewed the existing cryopreservation protocols for human ovarian tissue and found that the following combinations of non-permeating and permeating cryoprotective agents are used worldwide: DMSO alone, DMSO in combination with sucrose, DMSO in combination with 1,2-propandiol, and DMSO together with human serum albumin (HSA). There are also groups, which do not use DMSO in their protocols, but prefer the following combinations: 1,2-propandiol with sucrose or ethylene glycol with sucrose. None of the protocols is stated to be superior to the others for OTC (80).

## Different techniques of cryopreservation

In the field of ovarian tissue cryopreservation, currently, two different freezing methods are used: slow freezing and vitrification. The following paragraphs provide a detailed description of slow freezing and vitrification as methods for cryopreserving ovarian tissue and compare their advantages and disadvantages.

### Slow freezing

The slow freezing method for ovarian tissue, described already by Gosden in 1994, is a well-established method for cryopreservation but needs expensive equipment and is time consuming (82). The temperature changing rate during cooling and warming is a key factor for successful cryopreservation, thawing and fertility preservation. Slow freezing is the most commonly used method for OTC via different protocols, which are mostly modified after Gosden et al. (82). Freezing protocols can vary in the starting temperature [ranging from room temperature (RT) to 0 °C] and the initial cooling rate (5 °C/min to 0.5 °C/min), as they are not considered as main critical steps for tissue viability (80). However, the cooling rate after seeding differs. Isachenko et al. were the first in 2012 to use autoseeding in their slow freezing protocol (83). Almost all protocols employ a cooling rate of 0.3 °C/min until the temperature reaches −40 °C. The ice crystals grow at a defined rate, progressively increasing the concentration of the cryoprotectant in the tube. The cryoprotectant gradually penetrates through the tissue in a controlled manner. Fluctuations during this critical freezing process can cause irreparable damage to the tissue. The protocols also share the last step with a cooling rate of 10 °C/min until −140 °C. These steps are performed in an automatized freezer and are highly reproducible (19, 28, 31, 84–99). In addition to the differences in the freezing protocols themselves, there are also differences in the used cryoprotectants and the equilibrium steps. DMSO is frequently used at a concentration of 1.5 M or 10% - either alone or in combination with 0.1 M sucrose (19, 88, 91, 94, 98). But there are also published protocols using 12.5% DMSO (90) or DMSO in combination with PROH or HSA (85, 89). In some protocols DMSO is not used at all but PROH or EG in combination with 0.1 M sucrose was chosen (61, 87, 92, 96). Moreover, slow freezing protocols also differ in the equilibration of the tissue strips. In some protocols, this step is performed on ice for 15 min (94) whereas others equilibrate for 30 min on RT (92). So far, there is no consent on a standard protocol for slow freezing, making a comparison of published results quite difficult. Therefore, the question arised is, which protocol provides the best outcome for the patients.

### Vitrification

In contrast, vitrification, which was established more recently as cryopreservation method, is cheaper, less time consuming and has gained more and more importance (64). It provides a much shorter freezing protocol compared to the slow freezing process. Vitrification depends on adding high concentrations of CPAs (up to 40%) for accelerated dehydration, followed by placing samples directly into liquid nitrogen to immediately reach cryogenic status and stop all metabolic activities (78, 100). This rapid supercooling rate does not allow the water molecules to arrange into ice crystals. Instead, the intracellular water converts into an irregular amorphous glass-like solid (101–103). The word “vitrification” describes this process of becoming a glass-like structure by cooling (104). Vitrification not only avoids ice crystal formation while cooling down, but also shortens the exposure time to osmotic pressure, dehydration and CPA toxicity during freezing. Moreover, it reduces time, effort and costs, compared to the slow-freezing method (78).

Despite being highly effective in freezing single cells, e.g., sperm (105), oocytes (106) and pancreatic islets (107), as well as simple structured tissues and cell clusters, e.g., early embryos (108), vitrification is not yet well and routinely established for cryopreserving complex tissues like ovarian tissue. In complex tissues, CPAs require much longer time to penetrate through the tissue layers until they reach every single cell and also require longer time for cooling the entire tissue, which contradicts with the main principle of vitrification to minimize the dehydration exposure time. Another important reason is that complex tissues are composed of different cell types, which have different sizes, surface areas, intercellular connecting features and permeabilities and accordingly, require different individually optimal cryomedium compositions and cooling rates (109). Nevertheless, researchers continuously keep trying to apply vitrification on larger tissue samples, e.g., ovarian tissues. Vitrification of ovarian tissues showed less DNA damage in primordial follicles and more stromal cell survival compared to slow freezing (110) and promising restoration of reproductive functions after reimplantation (111). Live births resulting from retransplantation of vitrified ovarian tissue increased worldwide from 2 births in 2015 published by Suzuki et al. (30) to 4 in 2018 reported by Silber et al. (26) and to 6 births in 2024, when Saenger et al. and Keros et al. (40) published the first successful delivery in Europe (39). However, ovarian tissue vitrification is not yet commonly used, as its protocols still need to be improved. Because vitrification requires the addition of high concentrations of CPA, which exhibits toxicity on cells, many vitrification protocols tend to add combinations of different CPAs, in order to milden the specific toxicity of each solely used CPA (112–114). Similar to the slow freezing protocols, there are also differences in the vitrification protocols. One main difference is the carrier system used. Suzuki et al. (30), Silber et al. (26) and Saenger et al. (39) used metal based carrier systems, whereas other groups used plastic based carrier systems (115). In addition to these two carrier systems, several other materials have been tested, like quartz microcapillary (116, 117), electron microscopy grids (118) and gel loaded tips (119, 120). Within the groups who used metal based carriers, the vitrification protocols also differ in the use of cryoprotectants. Suzuki et al. and Saenger et al. used EG in different concentrations in combination with Serum Substitute Supplement (SSS), polyvinylpyrrolidone (PVP) and sucrose, whereas Silber et al. vitrified with EG in combination with DMSO, SSS and sucrose (26, 30, 39).

There are efforts to harmonize the protocols for slow freezing and vitrification by organizations like the FertiPROTEKT network, the Oncofertility Consortium, ESHRE, ISFP and the global initiative OvaNet. The aim is to identify the most successful protocols, evaluate the best combination of handling, storage conditions, and transport, and additionally to educate staff to further improve the overall results for the patients (42, 121–123).

### Comparison of slow freezing and vitrification

An important challenge for comparing the results of slow freezing and vitrification is that researchers cannot compare the ovarian follicle numbers before freezing and after thawing within exactly the same sample (29). The most appropriate approach to compare these methods is a split sample design, like it was performed by Schallmoser et al. (124, 125) determining follicular viability via Calcein-AM staining. The findings reported by Schallmoser et al. (124, 125) as well as the results of Suzuki, Keros, Sugishita, Silber and Nikiforov, demonstrated, that vitrification is a promising technique for cryopreservation of ovarian tissue (26, 30, 113, 126, 127), as it offers several advantages over conventional slow freezing methods which were already published by Amorim et al. in 2011 (128). In comparison to slow-freeze media, vitrification media are commercially available. Therefore, quality standards can be better maintained and variability in self-made media production can be avoided. Furthermore, vitrification does not need an expensive freezer (Table 1). The functionality of slow freezing is supported by >200 live births after retransplantation, whereas live births after retransplantation of vitrified ovarian tissue are limited (30, 39, 59, 111). In 2017, 14 non-randomized studies were analyzed comparing slow freezing and vitrification as OTC methods with the conclusion that vitrification might be more effective in preserving ovarian tissue. However, Shi et al. noted variabilities in the vitrification protocols and the need for further validation through prospective randomized studies (110). A recently published study of Le et al. compared the viability of slow frozen and vitrified ovarian tissue before and after transplantation. Comparison with fresh tissue highlighted that any kind of cryopreservation compromises follicle development, follicle survival and their function as well as the vascularization of ovarian tissue. Direct comparison of slow frozen tissue with vitrified tissue revealed a significant higher number of viable follicles in slow frozen and transplanted tissue compared to vitrified and transplanted tissue. As proliferation and neo-vascularization of slow frozen and transplanted tissue were also better in comparison to vitrified and transplanted tissue, slow freezing was anticipated as preferred cryopreservation technique in these cases (129).

**Table 1:** Comparison of slow freezing and vitrification methods in OTC highlighting the advantages and disadvantages of both methods.

Method	Advantages	Disadvantages	References
Slow freezing	- Low concentration of cryoprotectants - Highly reproducible freezing process (biofreezer/ programmable freezer)	- Intracellular ice crystal formation - Expensive equipment - Non-uniform cooling gradient between periphery and core of tissue - Time consuming	Meirow D et al., 2001 (84)
Vitrification	- No internal ice formation - Reduced exposure time to osmotic stress, cryoprotectant and dehydration - Less primordial follicular DNA damage - Vitrification medium commercially available - Cheaper - Faster	- High concentrations of cryoprotectant - Less established method	Chen H et al., 2022 (78) Behl S et al., 2023 (130)

## Warming

Freezing ovarian tissue is only one part of the process, as the tissue has to be thawed again before retransplantation. Even though the CPA reduces the damage during freezing, it can cause a lot of problems while thawing the tissue. The risk of ice crystal formation exists regardless of the freezing method (80), therefore the interplay between cooling and warming rates must always be carefully considered. For vitrified tissue, the risk of recrystallization during warming is more serious than for slow frozen tissue. One reason for this is the high concentration of CPAs used in the vitrification process (131). The removal of the CPAs is therefore one of the most critical steps while warming due to the osmotic imbalance that may occur due to water uptake (80). In line with the variations in the cryopreservation protocols, the published warming protocols vary a lot in terms of temperature, time and performed steps as well. Some protocols keep the cryopreserved tissue at RT (86, 91, 97), before transferring them to 37 °C, whereas other protocols start the thawing process directly at 37 °C (89, 93, 95, 132–134). After this thawing step, which most of the protocols share, some protocols suggest transferring the tissue back to 4 °C for washing and diluting the cryoprotectant (91, 133). For the dilution steps, the protocols use decreasing concentrations of the cryoprotectant, mixed with either sucrose, Roswell Park Memorial Institute (RPMI) media, minimum essential media (MEM) with autologous serum or Leibovitz medium. The only component many protocols have in common is sucrose, which is used in different concentrations at some point during the dilution of the cryoprotectant (86, 89, 91, 93, 95, 97, 133, 134).

### Removal of cryoprotective agents

Removal of the cryoprotective agents, while thawing the tissue, is an additional critical step, where viable cells may be lost. Thawing the tissue might cause the cells to swell due to osmotic imbalance caused by the used CPAs. To reduce the risk of cell swelling, the CPAs need to be removed slowly and controlled with support of sugars (e.g., glucose or sucrose) or proteins, like HSA or a serum substitute supplement. In addition to sugars or proteins, solutions with lower CPA concentrations are applied during CPA removal (80). Nevertheless, during the CPA removal process, the exposure to CPAs has toxic effects on the tissue. A possibility to reduce the need of traditional CPAs like DMSO or Ethylene glycol is to address the mechanism of cryopreservation-induced delayed onset of cell death (CIDOCD) (135–137). This mechanism is a stress response of cells activated during the cryopreservation process while freezing and thawing (135). Different research groups have shown that by controlling this stress response, cell survival and cell functions were improved (138–140). The discovery of this stress response and possibility of its modulation resulted in a rethinking of cryopreservation. Today, this approach is already implemented into commercial intracellular-like cryopreservation media like CryoStor®. Nevertheless, there is need for further improvements regarding continued cell loss and impaired function in complex tissue post-thawing (141).

### Thawing and rapid warming

Although vitrification minimizes injuries during freezing, it does not prevent tissue injuries during warming. As conventional warming rates are not as rapid like the vitrification cooling rate, they allow devitrification, which is “the formation of ice crystals during warming” (142). This occurs if vitrified water molecules have enough time during slow warming to rearrange themselves into crystalline structures. Similar to ice crystal formation during slow freezing, devitrification also causes mechanical stress and disruption to the cells during warming (143). In addition, slow warming rates still allow long exposure to dehydrating conditions, cryoprotectant toxicity and oxidative stress before eliminating CPAs (Table 2). Within the scientific community, the slow warming protocols, which take 30 min or longer, have been used until 2016 (25, 144). To overcome the problems of devitrification during slow warming, rapid warming protocols were established by thawing the tissue within 10 to 15 min (1, 39). By shortening the warming time, the highly concentrated CPAs have significantly less time to damage the follicles in the ovarian tissue. Research in the field of rapid warming started already in the early 2000s, when Parmegiani et al. evaluated the follicle viability after the rapid warming protocol (145). Furthermore, research is currently underway to establish a universal rapid warming protocol, suitable for both vitrified and slow-frozen ovarian tissue. The first published results show equivalent or even superior results for the preservation of primordial follicles, when slow frozen ovarian tissue is thawed rapidly rather than using the standard slow warming protocol (145–147).

**Table 2:** Comparison of currently used thawing methods (slow warming, rapid warming) to methods under development aiming also to thaw larger tissue volumes.

Method	Advantages	Disadvantages	References
Slow warming		- Takes more than 20 min	Rivas Leonel EC et al., 2019 (80)
Rapid warming	- Takes only 10 to 15 min - Reduces exposure to high concentrations of CPA	- Only applicable for small samples - Risk of devitrification	Manuchehrabadi N et al., 2017 (148) Sänger N et al., 2024 (39)
Photothermal heating	- High remaining viability - High biocompatibility of Ti_3_C_2_T_x_ - Antibacterial ability	- Limited sample penetration => small sample sizes required	Panhwar F et al., 2018 (155) Alvarez C et al., 2022 (156) Cao Y-X et al., 2009 (157) Cao Y et al., 2022 (158)
Electromagnetic warming	- Larger samples can be rewarmed - High viability after warming	- Not applicable for human organ sizes, yet - No long-term viability studies available, yet	Manuchehrabadi N et al., 2017 (148) Han Z et al., 2023 (162)

However, there are limits to rapid warming protocols when it comes to the sample size and the size of the vitrified system. Rapid warming protocols do already fail to thaw tissues effectively in volumes of more than 5 mL (148, 149). Therefore, attempts are made to address this problem with support of magnetic nanoparticles (148) and lasers (150), which is explained below.

### Nanowarming

**Figure 3:**
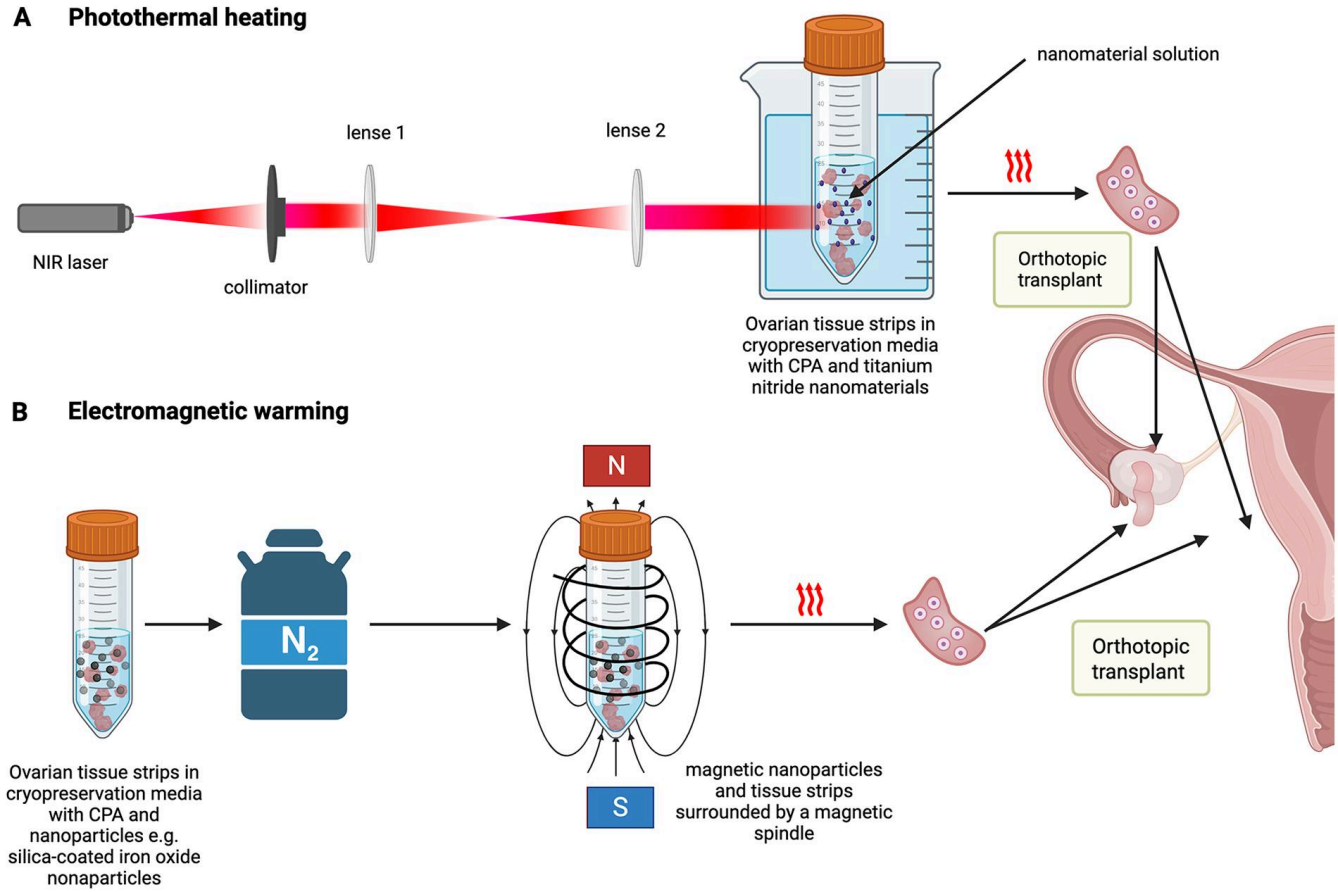
Nanowarming methods: **(A)** photothermal heating: method of light heat conversion by using photothermal nanomaterials during the cryopreservation process. The near-infrared laser (NIR) is used to transport greater energy to the nanoparticles and enhance the penetration depth. **(B)** Electromagnetic warming: addition of ferromagnetic nanoparticles and cryoprotective agent cocktail to the specimen. Heat for tissue warming is generated by exposure to an alternating current (AC) magnetic field. Silica-coated iron oxide nanoparticles (sIONPs) are used and washed-out following specimen warming. Created in BioRender. Eberhart, S. (2026) https://BioRender.com/y8p0uv8.

The idea of nanowarming—also known as volumetric heating—is that the vitrified systems can be larger than within the rapid warming protocols (151, 152). There are two different approaches in nanowarming: one method uses lasers (photothermal heating) and the other method uses a magnetic field (electromagnetic warming) to rewarm the samples (Figure 3).

### Photothermal heating

Photothermal heating is a method to rewarm cryopreserved biomaterials with near-infrared laser (NIR) and plasmonic nanomaterial which is already successfully in use in the mouse model since 2014 (153) (see Figure 3A). In other animal models, like zebrafish, the photothermal model using laser pulses was not promising (154). The photothermal heating was modified by adding nanodrops (for example gold particles) and a cryopreservation agent which leads to an increased viability in zebrafish embryos. Compared to gold nanodrops (GNRs), titanium nitride nanomaterials (TiN) lead to much better results with human cells (155, 156). The advantages of using TiN compared to GNRs is that the TiN have a better photothermal ability, provide increased heating rates and improve the temperature uniformity while laser warming. Another advantage is the high biocompatibility of TiNs which was published in 2022 by Cao et al., for cultivating human dermal fibroblasts (HDF) with a TiN solution for 24 h, resulting in a survival rate of 96% (157). By using Ti_3_C_2_T_x_—a two-dimensional transition metal carbide belonging to the MXene family—in warming cryopreserved cells, the devitrification effects were reduced and so were the effects of recrystallization. The antibacterial activity of Ti_3_C_2_T_x_ is another advantage to prevent contamination (158). Until now, this method of photothermal heating is only used for small samples as it shows limited sample penetration (150) (Figure 3A).

### Electromagnetic warming

Most electromagnetic warming protocols include the addition of ferromagnetic nanoparticles and a CPA cocktail to the specimen during the freezing process (Figure 3B). The heat for warming the tissue is generated by the exposure to an alternating current (AC) magnetic field (159). One type of the used nanoparticles is silica-coated iron oxide nanoparticles (sIONPs), which are washed out after the warming of the specimen (160). The remaining nanoparticle concentration in the specimen needs to be kept within tolerable limits, but the efficiency of the washing steps depends on tissue factors, like organ geometry and nanoparticle factors, for example size and shape (161).

Electromagnetic warming with magnetic nanoparticles started in 2017 and gave rise to the possibility to freeze and thaw tissue volumes up to 80 mL (148). Using this approach, a vitrified rat kidney was nanowarmed after storage of 100 days, transplanted and functioned then for 30 days post transplantation (162). This study hint at the direction in which the combination of vitrification and nanowarming could develop. However, further studies are necessary with organs, which are of comparable sizes to human equivalents, such as porcine organs. Furthermore, the possible toxicity of magnetic nanoparticles inside the tissue has to be considered (163), and the observation period needs to be extended to study long-term vitality and functionality (Table 2). Although limitations and challenges remain, the approach of vitrification has improved since 1984 (102); in combination with electromagnetic warming, it raises hope for further solutions of whole organ transplantation in the future (Figure 3B).

## Whole organ cryopreservation and retransplantation

One of the major challenges of freezing a whole human organ is the establishment of freezing protocols with a sufficient distribution of CPAs through the whole organ. There are animal studies working on different perfusion models, which are improving the CPAs distribution. So far, the best results in ovine (164, 165) and bovine tissues (166) have been obtained through the perfusion of an ovary for 60 min with a perfusion rate of 2.5 mL/min. The cryopreservation medium in these cases always contained DMSO as cryoprotective agent. Whole organ retransplantation of frozen and thawed human ovaries could give rise to the possibility of restoring the whole ovarian reserve *in vivo*. In this way, the loss of follicles after retransplantation of only ovarian tissue fragments, due to ischemic processes, could be reduced by re-perfusion of the whole ovary achieved through vascular re-anastomosis *in vivo*.

The procedure of whole organ cryopreservation with the slow freezing method and auto-transplantation have already resulted in several life births in sheep from 2006 to 2016 (165, 167, 168). Beside the challenge of distributing the CPAs through the whole organ, Hossay et al. (169) identified two additional challenges: the surgical skills, which are needed to retrieve the vascular pedicle, and the successful execution of the vascular re-anastomosis during the retransplantation.

Even though the procedure of freezing, thawing and retransplanting of a whole ovary was only successfully performed in animals (165, 167, 168, 170–182), there are publications about technical aspects which can be applied to humans, which have to be considered (183, 184). It has already been shown in rats, sheep, rabbits, dogs, monkeys and humans that an ovarian vascular transplantation is technically achievable. A successful retransplantation of a fresh human ovary of monozygotic twins was published already in 2008 which led to a successful pregnancy (27). Jadoul et al. defined two requirements which should be met to successfully perform an ovariectomy and whole ovary cryopreservation followed by retransplantation: preparation of sufficiently long ovarian pedicles, and as short as possible ischemic interval before cryopreservation and between thawing and retransplantation. To meet these requirements, Jadoul et al. suggested to remove the ovary through laparoscopic dissection (184).

In 2004, Martinez-Madrid et al. (183) published a protocol on how to freeze and thaw an intact human ovary with its vascular pedicle by using a passive cooling device - in this case a cryofreezing container. This publication gave a first idea on the viability of the follicles in a frozen and thawed whole human ovary, showing that the number of viable follicles was reduced from 98.1% to 75.1% after thawing. The ovaries were perfused prior to cryopreservation with Leibovitz L-15 medium containing DMSO and HSA. For perfusion, a pre-calibrated pump with a flowrate of 2.5 mL/ min was used. The follow up study of Martinez-Madrid et al. in 2007 investigated damages and the percentage of dead follicles in the perfused ovaries. The results were promising, as there were no Caspase-3 positive follicles found and the ultrastructure of the vessels was intact (185). The limitation of this publication is caused by the fact that the evaluation of Caspase-3 and the use of TUNEL assay do not give any information about the long-term survival (164, 178).

## Transportation, storage, long-term cryobanking and return rates

In the context of OTC, the following factors must be further investigated in the future, as they can significantly influence the quality of stored ovarian tissue and subsequent outcomes for patients. Key aspects include transportation conditions, storage and the effects of long-term cryobanking—particularly given that prepubertal ovarian tissue may be stored for more than ten years. Furthermore, the low return rates should be questioned.

Transport is especially relevant for facilities that offer ovarian tissue retrieval, but lack on-site cryopreservation and storage facilities. Therefore, the tissue is maintained at 4 °C in transport medium and transferred overnight to specialized and centralized cryobanks. Previous studies have shown that when ovarian tissue is transported at 4 °C, the number of primordial follicles remains stable for up to 21 h before apoptosis is initiated (186). These findings are consistent with data from the FertiPROTEKT network, showing that overnight transport does not impair pregnancy rates (187, 188) and confirm previous reports by Kristensen et al. and Schallmoser et al. (189, 190)*.*

However, storage conditions and long-term cryobanking in ovarian tissue cryopreservation remain poorly investigated, despite the fact that tissue from prepubertal girls may be stored for over ten years (61, 189, 191–196). Fabbri et al. reported in 2016 that ovarian tissue, cryopreserved under appropriate conditions by experienced personnel, can be stored for more than ten years (196). Ovarian tissue can be stored in the vapor phase of liquid nitrogen at −160 °C in automatically refilled tanks (190) or alternatively in the liquid phase. Large, automatically refilled liquid nitrogen tanks (up to 300 L) provide greater temperature stability than smaller containers but are associated with higher costs. This raises the question of whether centralized ovarian tissue cryobanking might be beneficial, particularly for long-term storage (197–199). It is currently unclear, whether long-term storage differentially affects ovarian tissue preserved by slow freezing vs. vitrification. Moreover, no data are available on the impact of carrier material (metal vs. plastic) on long-term storage outcomes following vitrification.

So far, reported low return rates include patients who do not survive cancer, patients who retain fertility after gonadotoxic treatment and conceive spontaneously, or patients who have not yet reached reproductive age or do not wish to become pregnant (200, 201). In a Danish cohort, Kristensen et al. reported a mean storage duration of 4.3 years, reflecting a mean age of 28.9 ± 7 years at the time of OTC, with 46% of unused tissue stored for more than five years (189). Recently, Yde et al. conducted a systematic analysis of English-language publications, also reporting low return rates (<10%) of ovarian tissue (195).

## Clinical applications

Many years often pass from the time of ovarian tissue retrieval to its retransplantation. Young women and girls facing imminent gonadotoxic therapies opt for the removal and cryopreservation of ovarian tissue, even though their desire to conceive may arise years or even decades later. As a result, patients, physicians and involved biologists learn about the success or failure of the retransplantation many years after the performed procedure. This stands in contrast to ART procedures. In fresh ART treatments, oocytes are retrieved, fertilized *in vitro*, and transferred back into the endometrium no later than 5 to 6 days after culture within the same cycle. The large amount of global patient data and the immediate feedback regarding successful pregnancy outcomes offer significant opportunities to refine protocols. Consequently, many pharmaceutical companies have entered this field in recent years and have developed and optimized media and cryoprotectants that have not only advanced the field but also proven to be lucrative.

In contrast, the long interval between the retrieval and retransplantation of ovarian tissue, combined with the significantly smaller number of patients and the reduced economic interest, has consequences on the development and refinement of new techniques. Nevertheless, vitrification has emerged as a key player in ovarian tissue cryopreservation in recent years, with established methods that even have resulted in spontaneous pregnancies following retransplantation. Similarly, the slow freezing method has been successful, offering various freezing protocols and cryopreservation media.

Despite the successes of those approaches, the preparation and cryopreservation of ovarian tissue differ significantly between them. In the slow freezing method, initial bovine studies demonstrate that ovarian cortex tissue segments with remaining medullary portions promote faster revascularization and thus reduce tissue atresia (202). Consequently, the tissue segments are relatively large, with a diameter of approximately 2 mm. Conversely, in vitrification, tissue strips are prepared to be thin and without medullary portions (≤1 mm in diameter) to enhance tissue integrity.

Additionally, a successful retransplantation of ovarian tissue not only depends on optimized cryopreservation and thawing protocols but also on skilled and experienced physicians and biologists. These professionals must expertly handle the retrieval, cryopreservation, thawing, and retransplantation processes to reactivate female fertility effectively. It is crucial to note that 80% of tissue damage occurs after transplantation (203). Tissue that does not establish vascular connections within the first few days after transplantation will be atrophic. Therefore, future revascularization must be carefully considered and optimized during tissue preparation processes.

## Overview of existing literature on ovarian tissue cryopreservation (OTC)

Over the past five years, there has been a substantial increase in published literature on ovarian tissue cryopreservation (OTC), with more than 100 reviews indexed in PubMed. While many of these studies address similar core topics, their thematic focus varies significantly. For example, Karimizadeh et al. presents a perspective aligned with the current review, emphasizing OTC as a cryopreservation technique while also exploring transplantation sites and their influence on the clinical outcome (199). In contrast, Bahroudi et al. compares slow freezing and vitrification methods, assessing their respective impacts on cell integrity, follicular quality, and apoptosis (111). Several reviews provide a broad overview of fertility preservation methods, offering general insights into indications, techniques, and outcomes (20, 204–207). Park et al. extends this work by comparing fertility preservation guidelines across different professional societies (208). A specific subset of the literature focuses on OTC in children, adolescents, and young adults (CAYA), highlighting age-specific considerations and clinical challenges (209–212). Regardless of their scope and impact, none of the reviews provide comprehensive information on ovarian tissue cryopreservation. Numerous reviews investigate OTC as a fertility preservation strategy tailored to specific patient populations. For instance, La Marca and Mastellari explore its role in patients with genetic conditions such as premature ovarian insufficiency (213). Cheng et al., Bollig et al., and van der Coelen et al. examine its application in women with Turner syndrome (214–216). The use of OTC in oncology is a significant area of interest: reviews by Lee et al., Moragón et al., Mahmood et al., Ulrich et al., Silvestris et al., and Salman and Covens focus on cancer patients, with particular emphasis on breast and cervical cancer subgroups (217–222). Jaeck et al. provides a detailed guide on fertility preservation strategies for various cancer types, based on current evidence (223). These reviews highlight the importance of OTC as a fertility preservation strategy for various patient groups and underscore the considerable variation in guidelines across different diseases.

In addition to disease-related indications, OTC is increasingly discussed in the context of social and elective fertility preservation. Reviews by Oktay et al. and Sacinti et al. evaluate the potential role of OTC in delaying menopause, while Varlas et al. and Kasaven et al. consider its use for social freezing (224–227). The relevance of OTC for transgender individuals has also been highlighted in recent reviews (228, 229). A number of recent publications address methodological advancements aimed at improving OTC outcomes. These include studies on alternative approaches to mitigate the risk of malignant cell reintroduction (230), primordial follicle activation (231), and various technical refinements (232–236). OTC is also widely featured in the context of ovarian tissue transplantation (OTT). Xie et al. discusses graft site selection (237), while Diaz et al. evaluates clinical parameters such as tissue fragment size and their influence on pregnancy and live birth rates (238). Najafi et al. addresses a persistent clinical challenge—follicular loss and the short lifespan of grafts—framing this issue within the context of oxidative stress, based on an analysis of 18 studies (239). These reviews emphasize, that successful fertility preservation depends not only on OTC but also on OTT. Several systematic reviews and meta-analyses compare OTC to other fertility preservation techniques, focusing on clinical outcomes such as pregnancy, miscarriage, and live birth rates (45, 240–243). As cryopreservation techniques have evolved, reviews have increasingly addressed the comparative efficacy of slow freezing and vitrification (111, 130, 244, 245). Marcantonini et al. further explores the role of various cryoprotective agents, with particular attention to the use of melatonin (73). Collectively, our review and this expanding body of literature highlights both the clinical relevance and the rapidly evolving methodological landscape of OTC across a wide range of indications and patient populations.

## Conclusion

Cryopreservation is routinely used to preserve cells and whole tissues, for example ovarian tissue. As OTC is an important option for female fertility preservation—especially in patients, where oocyte freezing is not applicable, e.g., for prepubertal girls or if there is not enough time before cytotoxic therapy—the need for optimized and efficient protocols with a high yield of living and physiologically functioning oocytes after thawing is desirable.

This review sheds light on the status quo of OTC and reveals that even though the protocols have been modified, many questions still remain to be answered. The ongoing research in this field reflects the existing need for improvement, but also for new innovative approaches to find solutions for the existing obstacles. In future, cryopreservation might revolutionize the field of organ transplantation, if whole organ freezing and thawing processes are able to reveal fully functional organs for transplantation. One of the major challenges is the establishment of freezing protocols with a sufficient distribution of CPAs through the whole organ. There are animal studies working on different perfusion models to improve CPA distribution. Whole organ retransplantation of frozen and thawed ovaries could give rise to the possibility of restoring the whole ovarian reserve *in vivo*. Thereby, the loss of follicles after retransplantation of only ovarian tissue fragments, due to ischemic processes, may be avoided by re-perfusion of the whole ovary achieved by vascular re-anastomosis *in vivo*.

In summary, a lot of progress has already been made in the field of tissue cryopreservation. However, there is still high potential for further improvements and optimizations of these methods. In case of OTC, this fundamentally increases the chances for women and girls to have their own biological children after severe gonadotoxic therapies. New innovative approaches applicable to humans open up new opportunities, not only in the field of fertility preservation but also for other tissues and whole organs.
